# Neural Stem Cells: Promoting Axonal Regeneration and Spinal Cord Connectivity

**DOI:** 10.3390/cells10123296

**Published:** 2021-11-25

**Authors:** Camila Marques de Freria, Erna Van Niekerk, Armin Blesch, Paul Lu

**Affiliations:** 1 Department of Neurosciences, University of California, San Diego, CA 92093, USA; errrna@gmail.com (E.V.N.); ablesch@health.ucsd.edu (A.B.); 2 Veterans Administration Medical Center, San Diego, CA 92093, USA

**Keywords:** spinal cord injury, neural stem cells, spinal cord connectivity, regeneration

## Abstract

Spinal cord injury (SCI) leads to irreversible functional impairment caused by neuronal loss and the disruption of neuronal connections across the injury site. While several experimental strategies have been used to minimize tissue damage and to enhance axonal growth and regeneration, the corticospinal projection, which is the most important voluntary motor system in humans, remains largely refractory to regenerative therapeutic interventions. To date, one of the most promising pre-clinical therapeutic strategies has been neural stem cell (NSC) therapy for SCI. Over the last decade we have found that host axons regenerate into spinal NSC grafts placed into sites of SCI. These regenerating axons form synapses with the graft, and the graft in turn extends very large numbers of new axons from the injury site over long distances into the distal spinal cord. Here we discuss the pathophysiology of SCI that makes the spinal cord refractory to spontaneous regeneration, the most recent findings of neural stem cell therapy for SCI, how it has impacted motor systems including the corticospinal tract and the implications for sensory feedback.

## 1. Pathophysiology of Spinal Cord Injury

Traumatic spinal cord injury is most commonly caused by compression, laceration and contusions which lead to severe impairment of motor/sensory function below the level of injury [1]. The magnitude of neurological symptoms, such as sensorimotor deficits, neuropathic pain, autonomic dysreflexia and bowel and bladder dysfunction, will depend on the level and severity of the trauma [2,3].

The initial traumatic event that may comprise fractures and/or dislocation of the vertebral column results in disruption of the vasculature, neurogenic shock and alterations in ion and neurotransmitter release. Ultimately this will set the stage for the secondary phase of injury [4,5,6,7] including post-traumatic ischemia, disbalance of neuronal electrolytes, accumulation of free radicals, glutamate excitotoxicity and inflammatory responses [8,9,10]. Activated resident microglia and monocyte-derived macrophages induce and magnify immune and inflammatory responses after the lesion [11,12]. Although some cytokines and growth factors secreted by microglia/macrophages can support neuronal survival, oligodendrogenesis, remyelination and angiogenesis [13,14,15,16,17], pro-inflammatory cytokines are detrimental for axonal growth and neuronal survival [18,19,20].

## 2. Detrimental Consequences after Spinal Cord Injury and Axonal Regeneration Failure

The lack of repair following spinal cord injury is due to both cell intrinsic factors and the extrinsic injury environment [21]. Neurons of the adult mammalian central nervous system (CNS) have a low intrinsic regenerative capacity due to a lack of growth-promoting signals, and the inability to activate the cellular machinery that enables the reestablishment of growth cones and axon elongation [22,23,24]. The injury environment also plays a key role in limiting functional repair after spinal cord injury.

**Figure 1:**
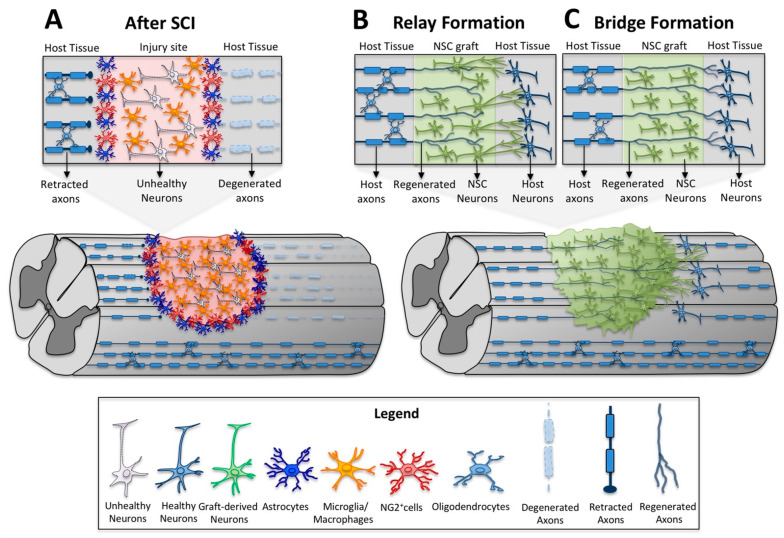
Repair of motor tract connectivity after spinal cord injury (SCI). (**A**) After SCI, activated microglia and hematogenous macrophages are recruited to the lesion site. Damaged descending axons die back from the lesion site and distal axons undergo Wallerian degeneration. Astrocytes become reactive and proliferate along the lesion border. Neural/glial antigen-2 positive cells (NG2^+^ cells) also proliferate and contribute to the scar at the lesion border. (**B**,**C**) Transplanted cells support and establish new connections across the site of injury through the formation of neuronal relays and/or cellular bridges. (**B**) To create a neuronal relay, grafted cells must: (1) survive and differentiate into neurons; (2) support growth of injured host axons into the graft; (3) make synapses with ingrowing host-derived axons; (4) extend axons into the distal host spinal cord; and (5) form synapses with host neurons below the lesion. (**C**) To create a cellular bridge: (1) host axons need to regenerate through the graft; (2) extend into caudal regions of host spinal cord; and (3) form synapses with host neurons below the lesion.

While the first traumatic damage leads to immediate and often serious impairment of neurological function, the secondary phase usually dictates the full magnitude of injury. Inflammatory cells and resident glia including astrocytes and oligodendrocyte progenitor cells become reactive at the lesion that culminates in a permanently remodeled tissue [25,26,27] (Figure 1). As a healing response, the formation of a scar acts to spatially isolate the wound and protect the surrounding spinal cord from further damage. However, a dense limiting border around the lesion core is formed and within and around the scar the formation and extension of growth cones is limited by a variety of inhibitory molecules [28]. These molecules can be broadly classified into two groups: myelin-associated inhibitors including NogoA, MAG and OMGp [29,30,31,32] and extracellular matrix (ECM) molecules including chondroitin sulfate proteoglycans (CSPGs), NG2, tenascin-C and Tenascin-R [27,33,34,35].

Another main component that limits axonal growth is the physical barrier formed at the lesion site. SCI usually results in a breakdown of the blood spinal cord barrier (BSCB) and vascular permeability [36]. Following BSCB disruption, a disbalance in ion flow leads to edema, blood vessel rupture and hemorrhaging that notably affect gray and white matter [37,38]. In mammalian SCI, a large zone of necrosis forms at the lesion site leading to cystic cavitation that prevents axonal growth across the site of injury [39]. Damaged axons fail to regenerate after injury, stop and neurons retract their axons once they reach the injury lesion border [28,40,41] (Figure 1A). The distal axon segment disconnected from the soma retracts from their postsynaptic inputs and undergoes Wallerian degeneration [42] (Figure 1A). SCI results not only in Wallerian degeneration of damaged axons, but also in demyelination of many remaining intact axons [43]. Attempts to promote axonal regeneration, structural plasticity and functional connectivity in this type of hostile environment is extremely challenging. Understanding the complexity of pathological events resulting from the initial trauma and the factors that limit regeneration of the damaged CNS allows for the identification of new therapeutic approaches that may improve sensorimotor outcomes.

## 3. Therapeutic Approaches to Overcome Obstacles in the Lesion Core and Promote Regeneration

In efforts to increase axonal regeneration and recovery of function, experimental approaches targeting the environment at the injury site such as inhibiting molecules associated with scar and myelin debris [29,31,44] have been the focus of many previous studies. Means to target the intrinsic growth potential, such as modulating the expression of PTEN and SOCS3 [45,46,47] have also shown some promise. In addition, a variety of pharmacological strategies to modulate the inflammatory response and to provide neuroprotection at early stages post-injury aim to reduce the long-term damage of the secondary injury [48,49,50,51]. Although these studies have shown some beneficial effects, they do not lead to robust regeneration of supraspinal axons into the lesion site or for long distances beyond a site of SCI.

One strategy to restore functional connectivity between severed spinal cord segments is the transplantation of neural progenitor cells (NPCs). NPCs have high therapeutic potential for reconstruction of the injured spinal cord tissue, since they can proliferate and differentiate into neurons and glia [52,53]. In addition, transplanted NPCs have been reported to exert plastic changes by remyelinating spared axons, facilitating chemotaxis after lesions, promoting neurite outgrowth and reducing cell death and axonal dieback [54,55]. These changes work in a synergistic manner to facilitate plasticity and regeneration of the injured spinal cord after cell transplantation. In adult rats, neuronal and non-neuronal cell transplants obtained from fetal and adult donors have been used to replace lost or impaired neurons and glia for almost 50 years [56,57,58,59,60,61,62]. In the 1970s, studies using transplantation of cerebellar and non-cultured neocortical tissue from adult donors into the lesion cavity in a canine spinal cord injury model showed no survival and no graft–host tissue integration [60,61]. A subsequent study grafting embryonic tissue from distinct neural areas into a partially and completely transected adult spinal cord showed modest tissue integration and axon growth of neocortical embryonic transplants within the host spinal cord [59].

A major issue in these earlier techniques was a lack of retention of neural tissue within the injured spinal cord parenchyma that resulted in poor cell survival and failure of axonal growth. Later studies successfully overcame this issue by providing a more appropriate source or substrate for axonal growth. An experimental model using a peripheral nerve inserted into the lesion in the CNS showed extensive growth of axons from transplanted neurons along the “neuron-free conduits” [63]. Later, a combination of two different transplantation methods of fetal neuronal and peripheral nervous system grafts showed that neurons in the graft not only survived but also extended axons from the superior colliculus to the striatum [62].

Although experimental studies in the 1980s showed a gradual improvement of cell survival, integration and axonal growth, there was no evidence of functional improvements. Only in the 1990s did intraspinal fetal grafts placed into the site of SCI in adult and newborn rats show some improvements in functional outcomes [57,58,64]. This partial restoration of supraspinal control and functional recovery was related to the formation of new neuronal relays bridging the injury [58]. Follow-up studies almost 20 years later using new tools and techniques supported the functional connectivity between NSC graft and host neurons by transneuronal tracing (pseudorabies virus) and electrophysiological recording [65,66]. Further studies using stimulus-evoked c-Fos expression and electrophysiological recording showed that host axons formed functional neuronal relays with graft neurons at the injury site [67].

The concept of neuronal relays is applicable when new neural circuits support and establish new connections across the site of injury. Specifically, descending host motor axons that project into stem cell grafts [68,69,70] establish functional synapses with graft-derived neurons, which in turn project axons into the caudal host spinal cord [71,72,73] (Figure 1B). Cellular grafts may also serve as bridges for injured axons that regenerate across the lesion site with appropriate stimuli (Figure 1C). Good evidence for cellular bridging across the lesion site typically requires complete SCI transections or complete transection of specific tracts to ensure that all descending and/or ascending tracts have been fully axotomized [74,75,76]. While several examples of cellular bridging have been reported, it remains challenging to induce a significant number of axons to exit the cellular bridge at the distal interface, in particular with more extended lesions.

## 4. Neural Stem Cell Transplants as a Relay for Spinal Cord Connectivity

As previously discussed, SCI usually results in a large lesion area that damages not only the central gray matter, but also the surrounding white matter axon tracts that connect the brain with the rest of the body. Although damage of local spinal cord gray matter neurons primarily affects local segmental functions, disruption of white matter axon tracts is the major cause of neurological deficits below the level of injury [77,78]. Unlike tissue in the peripheral nervous system, the CNS does not repair itself effectively [79].

Neural stem/progenitor cell transplants have the potential to reconnect damaged axons since transplant-derived neurons can function as neuronal relays for disrupted pathways. First, transplant-derived neurons can serve as new neuronal targets attracting injured host axons that regenerate into and innervate the graft. A typical example is the robust regeneration of corticospinal (CST) axons into neural progenitor cell grafts [69]. Second, transplant-derived young neurons have the intrinsic capacity to extend axons into the host parenchyma to form new synaptic connections. Studies with rodent and human cells have consistently shown robust and long-distance growth of transplant-derived neurons in different models of SCI [71,72,73,80]. The ingrowth and connectivity of regenerated axons with transplant-derived neurons and the outgrowth of transplant-derived axons and synapse formation with host neurons provide the basis for neuronal relays that re-connect proximal injured axons with spinal cord neurons distal to the injury site (Figure 1B).

**Figure 2:**
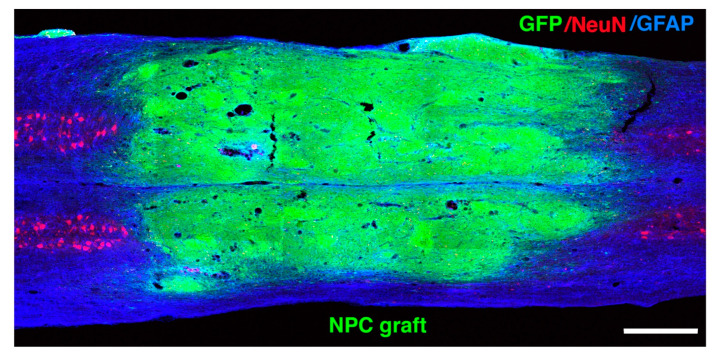
Transplanted neural stem/progenitor cells survive and completely fill a large and severe lesion site after a T-10 rat spinal cord contusion injury one-month post-grafting. GFP (green), NeuN (red), and GFAP (blue) triple labeling shows complete filling of the lesion cavity by GFP-expressing embryonic day 14 spinal cord-derived multipotent neural progenitor cells. Scale bar: 500 μm.

In order to successfully generate neuronal relays by transplanted neural stem/progenitor cells in the injured spinal cord, a continuous transplant that completely fills the lesion site is needed. Excellent tissue integration with the surrounding host parenchyma and direct contact of transplants with the host tissue are additional pre-requisites for the ingrowth and outgrowth of axons [69]. Large and severe injuries usually result in an inhospitable environment for stem/progenitor cell survival leading to inadequate integration and incomplete filling of the lesion site. A fibrin matrix containing a growth factor cocktail has successfully been used to retain transplanted cells in the lesion site and to support their survival, differentiation and integration [72,81,82]. Such an approach results in good survival and consistent filling of large lesions by transplanted neural stem/progenitor cells after severe SCI (Figure 2).

As an alternative approach, neural stem/progenitor (NSC/NPC) cells have been transplanted into remaining spared portions of the spinal cord, rostral and caudal to the lesion epicenter rather than in the lesion epicenter itself [83,84]. Most of these studies aim to re-myelinate spared axons by transplantation of oligodendrocyte progenitor cells. Although transplanted cells survive better in the areas surrounding the lesion than in the lesion epicenter, this approach generally fails to fill up large lesions and cannot serve as a bridge in the completely injured spinal cord.

In order to function as neuronal relays, a large proportion of transplanted NSC/NPC must differentiate into neurons that can reconnect injured circuits. Freshly dissociated rodent embryonic spinal cord-derived neural stem/progenitor cells usually differentiate into ~30% neurons and ~50% glia when embedded in a growth factor cocktail containing fibrin matrix within 7 weeks after transplantation [72]. In contrast, differentiation of human neural stem cells takes substantially longer and cells primarily differentiate into neurons in the first few months followed by glial differentiation at later stages [80]. Thus, the proportion of neurons and glia may dynamically change over time. Some studies have shown preferred differentiation of transplanted human neural stem/progenitor cells into glia after SCI. This may be related to cell culture conditions or the differentiation stage of neural progenitor cells as late-stage progenitors survive poorly and may have already differentiated into glia-restricted precursors.

For the formation of neuronal relays, propriospinal neurons and interneurons might be ideal as these neurons serve as natural “relays” in the intact spinal cord [85]. In preclinical studies, spinal interneurons and propriospinal neurons can serve as relays for injured circuits and promote functional recovery in incomplete spinal cord injuries [86,87]. One class of interneurons, V2a interneurons, are crucial for the transmission and coordination of motor and sensory function and can relay excitatory stimuli from the CST to the spinal central pattern generator that coordinates motor function. V2a neurons can be generated from rodent and human pluripotent stem cells [88]. Recent studies suggest that grafts of V2a interneurons in combination with NPCs can partially restore respiratory function in a moderate cervical spinal cord injury model. Although maturation of mouse ESC-derived V2a interneurons remained limited one-month post-transplantation, functional recovery was significantly greater in a subset of animals that received V2a-enriched NPCs compared to NPCs alone [89].

**Figure 3:**
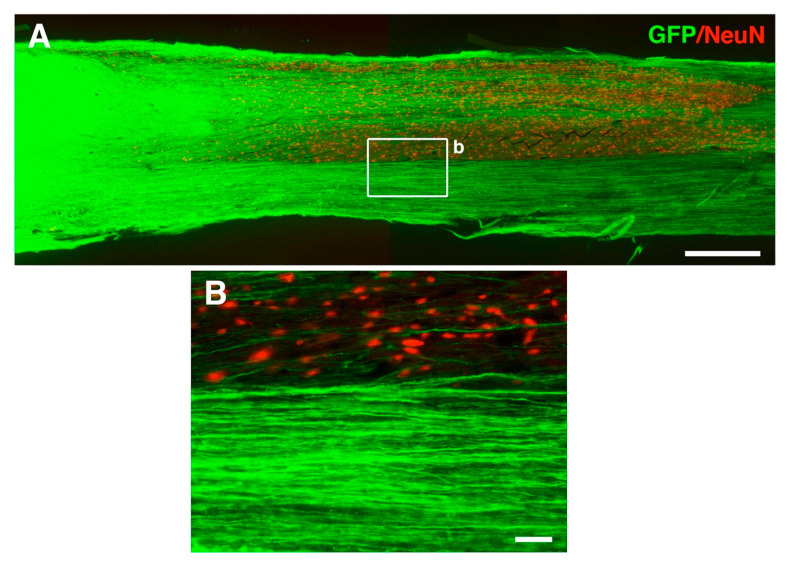
Robust axonal extension from human embryonic stem cell (UCSF4)-derived neural progenitor cells (NPCs) transplanted into a rat T9 crush injury site at 3 months post-injury. (**A**,**B**) Green florescent protein (GFP)+ human NPCs grafted into a T9 spinal cord crush injury in immunodeficient rats extend large number of axons caudally into host white and gray matter (sagittal section). Panel (**B**) is a higher magnification view of the boxed area in panel (**A**). Scale bar: (**A**) = 800 µm; (**B**) = 32 µm.

In general, transplant-derived neurons share many similarities with developing neurons. This includes a high intrinsic growth capacity allowing for axon extension into surrounding areas and remote regions. Transplanted neural stem/progenitor cells supported by fibrin-containing growth factors survive and differentiate into healthy neurons that can robustly extend their axons into host white matter over very long distances to innervate host gray matter. As many as 29,000 axons were shown to emerge from rat NPC grafts in caudal direction (0.5 mm caudal to a T3 complete transection) in the rat [72]. Graft-derived axons can travel for more than 20 mm (7 spinal segments) in rostral direction and 27 mm (9 segments) in caudal direction. Transplanted human neural stem cells can give rise to even higher number of axons (up to 150,000) extending into the host for even longer distances (the entire length of the rat CNS is around 100 mm) (Figure 3).

## 5. Emerging Therapeutic Interventions Using Human ESCs and iPSCs

The traditional source of neural stem/progenitor cells from the developing CNS contains various stages of NSCs and progenitor cells. Fetal CNS tissue, such as brain and spinal cord, can either be directly transplanted without prior dissociation [90], dissociated and transplanted as fresh neural stem/progenitor cells [72] or first expanded in vitro for subsequent transplantation [73]. Although NSC/NPCs derived from developing CNS tissue closely resemble developing neural stem/progenitor cells in vivo, the use of embryonic or fetal tissue faces ethical concerns and the tissue remains a limited resource.

An alternative means to generate neural stem cells or neural progenitor cells is the use of pluripotent stem cells including embryonic stem cells (ESCs) and induced pluripotent stem cells (iPSCs). ESCs are derived from the inner cell mass of the early blastocyst and have unlimited proliferative potential, therefore providing an unlimited resource of stem cells. In addition, ESCs are able to differentiate into a variety of cell types including neural cells [91]. iPSCs have similar characteristics as ESCs, but they can be derived from adult somatic cells, which allays ethical concerns associated with the use of human fetal/embryonic tissue. iPSCs may also reduce the risk of immune rejection of implanted cells since they can be generated from the patients’ autologous cells [92]. The generation of iPSCs has revolutionized regenerative medicine since these cells can not only be used for in vitro modeling of diseases and identification of novel therapies, but also for neural cell transplantation to replace lost neurons and glia in neurodegenerative diseases and CNS injuries.

In order to replace lost neurons and glia and to generate functional neuronal relays for CNS repair, pluripotent stem cells need to be induced to differentiate into neural cell lineages, especially to neural stem/progenitor cells or even early-stage neurons through a process called neural induction and differentiation. Early studies used a method of embryoid body (EB) formation and manual selection of neural rosettes after spontaneous differentiation or neural induction using growth factors or co-culture with PA6 stromal cells. Recent studies have focused on manipulation of specific signaling pathways such as inhibition of BMP, TGF beta (also called dual SMAD inhibition) and WNT pathways by proteins and/or small molecules for neural induction. In addition, the early stage neuroectoderm can be patterned by different morphogens to produce distinct rostro-caudal, dorsoventral and mediolateral neurodevelopmental lineages [88,93].

Interestingly, in caudal spinal neurodevelopment, neural progenitors are derived from transient neuro-mesodermal progenitors that are capable of generating both spinal cord neural and mesodermal progenitors [94]. This principle of spinal cord development can also be used to derive spinal cord neural stem cells from human pluripotent stem cells. When these homotypic ESC- or iPSC-derived neural stem/progenitor cells are successfully transplanted into the injured spinal cord, they differentiate into both neurons and glia, extend a large number of axons into the surrounding spinal cord and attract host axon regeneration into the graft [71,93]. This is significant, because previous studies have shown that rat neural progenitor cell (NPC) grafts of spinal cord identity support regeneration of the corticospinal tract (CST), a motor system most refractory to previous regeneration efforts [69]. In contrast, when NPC grafts originating from the embryonic rat telencephalon were placed into sites of SCI, the cells did not support regeneration of the CST motor projection, suggesting entopic grafts are crucial to the success of robust regeneration and functional recovery [69,72].

Additional studies with human ESC- or iPSC-derived neural stem/progenitor cells in animal models of SCI are needed to support the safety, feasibility and potency of these cells for clinical translation.

An alternative approach and short-cut is direct conversion of somatic cells into induced neural stem cells (iNSCs) [95,96]. Interestingly, these iNSCs are highly responsive to regional patterning cues and can differentiate into midbrain dopamine neurons and spinal cord neurons [94]. In addition, despite the original cell source (adult somatic cells), iNSCs may not inherit age-related DNA methylation patterns (depending on the protocol), indicating that adult somatic cells can be rejuvenated. Whether these cells are equipotent to ESC- and iPSC-derived NPCs in severe models of SCI remains to be determined in more detail.

## 6. Motor Axon Regeneration and Relays in Neural Stem Cell Transplantation

Descending motor tracts represent key elements to voluntary motor control in humans. One such pathway, the corticospinal tract (CST), commands its voluntary motor control not due to its connections with the motor cortex, but its immediate and direct access to spinal and motor interneurons within the spinal cord [97]. In humans, highly skilled fractionated movements elicited through the hand and fingers depend on the CST tract due to its direct cortico-motoneuronal connections within lamina VII, VIII and IX of the contralateral spinal grey [97,98]. When these direct connections are severed such as in a spinal cord injury, permanent motor deficits occur with no spontaneous regrowth of the CST tract and no restoration of CST-dependent motor function. Much attention has been given to the CST tract to restore some motor control including removing myelin from the extracellular milieu [99], neutralizing inhibitory glial proteins [100,101], enhancing trophic support in the injured environment [102,103], grafting cells within the lesion cavity [104,105], or enhancing the intrinsic growth state of the neuron [47,106,107,108,109]. While these approaches are very meritorious, they do not lead to robust CST regeneration into a lesion cavity, a critical component to restore cortico-motor function after severe injuries. The failure to elicit corticospinal regeneration into severe SCI lesion sites is a substantial limitation for the development of translational therapies for SCI.

**Figure 4:**
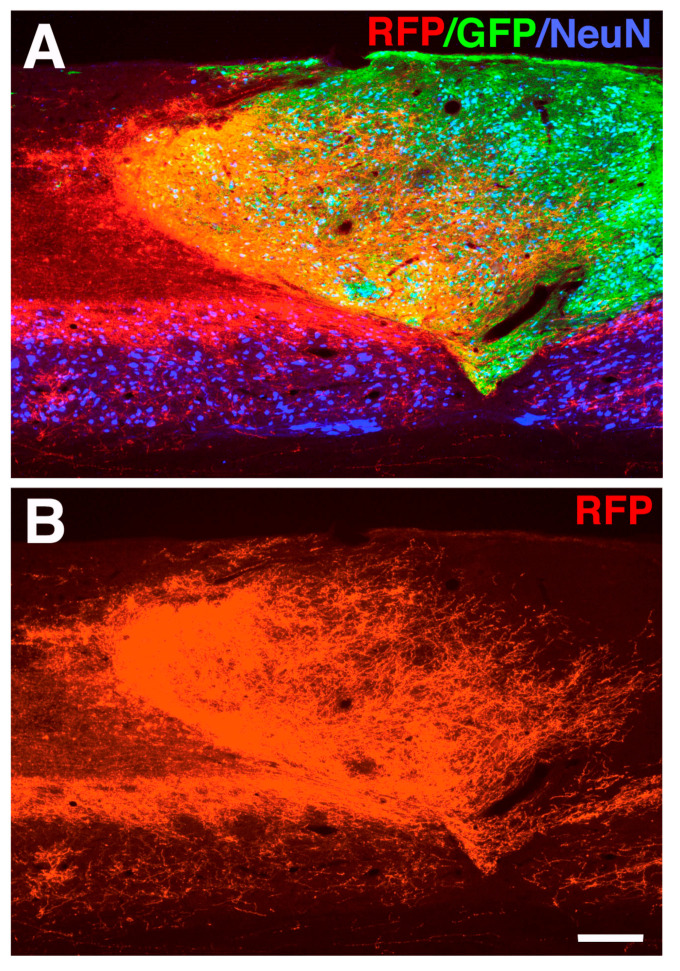
Robust regeneration of corticospinal tract (CST) axons into an E14 rat neural progenitor cell (NPC) graft after spinal cord injury (SCI). (**A**,**B**) Red florescent protein (RFP) labeled CST axons regenerate into a green florescent protein (GFP)-expressing NPC graft placed into a rat C4 dorsal column transection site that completely transects the main CST tract. Cells were grafted one week post injury and animals survived 6 weeks post-transplantation. Sagittal section, rostral is to the left, caudal to the right. NeuN (blue) in (**A**) labels both host and graft-derived neurons. Scale bar: 250 µm.

**Figure 5:**
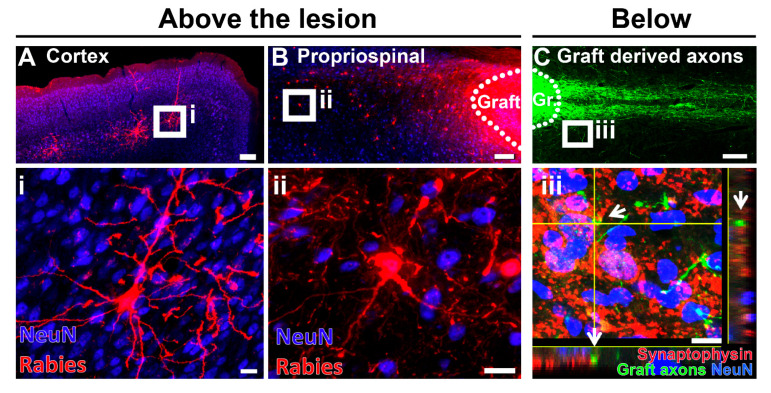
Neural progenitor cell grafts placed into a spinal cord lesion cavity form connections with host circuitry above and below the site of injury. Synaptic connections above the lesion: (**A**) Using a retrograde rabies-mCherry virus injected into the graft, monosynaptic connections with graft-derived neurons result in corticospinal motor neurons positive for rabies virus (red). (i) Inset shows high magnification of CST neurons positive for rabies virus (red), NeuN (blue). (**B**) Other neural circuits also form synaptic connections with the graft (dotted line). Reticulospinal neurons (not shown) and propriospinal neurons above the lesion within the spinal cord are positive for rabies virus (red). (ii) Inset shows high magnification of rabies positive (red) propriospinal neurons within the spinal grey above the lesion, NeuN (blue). (**C**) Synaptic connections below the lesion: Graft-derived axons (green) extend into the host spinal cord below the lesion. Graft indicated by dotted line. (iii) Inset shows graft-derived axons (green) forming synapse-like contacts with NeuN (blue) positive neurons within the caudal host spinal cord. Synaptophysin (red) indicates synaptic connections, arrows show GFP-positive grafted axons co-localizing with synaptophysin (red). Scale bar (**A**,**B**) = 250 µm; (**C**) = 100 µm; (i) = 20 µm; (ii) = 20 µm; (iii) = 10 µm.

To address this, we and others have used neural progenitor cell transplantation into a lesion cavity to provide a fortuitous substrate for axon regeneration and reinnervation [69,104,110]. Most notably, when primary NSCs of spinal cord identity were grafted into a severe spinal cord lesion, robust CST axon regeneration into the lesion site was observed and newly regenerating CST fibers made synaptic connections with grafted embryonic neurons [69] (Figure 4). Taking advantage of a modified monosynaptic rabies virus system, a retrograde rabies-mCherry virus was used to selectively infect graft-derived neurons. Transynaptic spread resulted in robust retrograde labeling of CST neurons within the primary motor cortex [70] (Figure 5). In separate experiments, after infecting host CST neuron with trans-synaptic herpes virus (HSV), which is anterogradely transported, HSV-positive neural cell bodies were identified within the transplanted graft [111]. These HSV-positive cells in the graft displayed characteristics of lower motor neurons including alpha motor neurons of the spinal cord (Chx10 positive cells) and motor synergy encoder neurons (Satb1 positive cells). This is consistent with previous observations that CST axons specifically innervate phenotypically appropriate motor interneurons within the graft [111]. Additional evidence for the functional integration of regenerated axons and graft-derived neurons was demonstrated by the activation of an abundance of embryonic grafted neurons within the lesion cavity in response to CST neuron stimulation within the motor cortex [112]. Interestingly, grafted neural progenitor cells receive extensive synaptic inputs not only from CST projections, but an array of host neural circuits including subcortical circuits such as the reticular formation, propriospinal neurons, rubrospinal projections and sensory inputs (Figure 5) [70,113]. This suggests that NPC grafts are also integrated into other motor and sensory circuits that may provide a substrate for functional relay formation [70,113].

This concept of neural relays was further supported when rhesus monkeys with a cervical spinal cord injury received a human caudal neural progenitor cell graft and showed CST axons growing into the graft for up to 500 µm [73]. Other axonal populations including serotonergic fibers and intraspinal neuronal circuits also extended into the graft.

Interestingly, more modern techniques have revealed the molecular signature of the adult corticospinal neuron as it regenerates after injury. Surprisingly, translational profiling indicated that during an early time after injury, CST neurons reverse back towards an immature developmental state and take on a gene expression profile similar to embryonic day 18 CST neurons [114]. This profile was sustained throughout active regeneration, similar to observations of other neuronal systems where a coordinated sustained gene expression profile is necessary for successful regeneration [115]. Interestingly, this genetic program was activated in the absence of a fortuitous cellular substrate within the lesion cavity early after injury, but over a period of days was lost and eventually downregulated when no stem cell substrate was present. Indeed, other developmentally important regulators including PTEN [109] and SOCS3 [108] have shown great promise in promoting regeneration and sprouting when downregulated within the CST neuron. Other molecular regulators will likely emerge as our methods become more sophisticated and, to date, partial regeneration or sprouting of the CST has been seen with the overexpression of KLF7 [107], STAT3 [116] and Sox11 [117]. Exploring further molecular signatures that control CST regeneration including epigenetic and proteomic signatures may yield novel insights into the CST neuron’s capacity to regenerate and establish new circuits.

One might ask if this strategy is feasible to establish the primordial circuits of the CST required for appropriate interneuron and motoneuron innervation within the spinal cord, leading to restored cortico-motor circuits for precise motor control. In the developing CST circuit in infant rhesus monkeys, CST fibers terminate within the intermediate zone of the spinal grey and no CST fibers innervate the motoneuron pools of the ventral horn [97]. During this time, one-month-old rhesus monkeys cannot execute independent skilled finger movements and fail to remove morsels of food from small food wells with a pincher movement of the index finger and thumb [118]. This skill gradually develops over the next 6–8 months of age, during which time the CST fiber terminals begin to innervate the motoneuron pools of the ventral horn in the cervical spinal cord [97]. Thus, with repetitive behavioral movements, training-induced adaptation of the CST tract leads to precise innervation of motoneurons within the spinal cord that directly contributes to relatively independent finger movements. Investigating the CST tract of cebus and squirrel monkeys further supports this notion. Both of these non-human primates have pseudo-opposable thumbs, but these two primates differ markedly in their ability to manipulate small objects [119]. Only cebus monkeys have the ability to remove food with a skilled forelimb pincher movement where they can grasp and manipulate small objects using their index finger and thumb. Squirrel monkeys are incapable of performing skilled finger pincher movements and instead use a power grip where all fingers move together pressed against their palm in a sweeping motion to grasp an object [119,120]. Interestingly, the cebus monkey has abundant CST terminals within the ventral horn of the cervical spinal cord. In contrast, comparable CST terminals within these regions are absent in the squirrel monkey cervical spinal cord with the majority of CST fibers residing within the intermediate zone of the spinal grey [98].

Given that modern tools of neuroscience have unveiled that adult regenerating CST neurons regress back towards an embryonic fate while undergoing active regeneration, one can hypothesize that activation of this intrinsic developmental program can be harnessed to drive new CST circuit formation, and that in combination with training-induced adaptation, projections towards the appropriate targets within the spinal cord can be achieved.

## 7. Sensory Axon Regeneration and Relays in Neural Stem Cell Transplantation

Most studies examining NSC-mediated behavioral improvements and formation of novel circuits have focused on descending motor systems, whereas restoration of sensory function from levels below a site of SCI has not received the same attention. Without doubt, there is a good rationale to focus on motor systems and specifically CST axons, one of the most important pathways for fine motor control. However, without any sensory feedback, precise supraspinal motor control will be difficult to achieve. Besides the important role of sensory feedback in motor control, regaining sensory function in areas below the lesion site could prevent or reduce some of the worst secondary complications from spinal cord injury including life threatening autonomic dysreflexia, detrusor sphincter dyssynergia that can lead to kidney failure, and pressure skin lesions, one of the leading secondary complications after SCI. In addition, pain at or below a site of SCI is highly prevalent in the SCI population, particularly in patients with incomplete injuries, severely impairing the quality of life [121,122]. Thus, NSC transplant-mediated recovery of sensory function or modulation of pain circuits are equally important targets for NSC-based therapies.

Ascending sensory projections include the dorsal column system, which is mainly responsible for fine discriminative touch and conscious proprioception. It is the only system with direct long primary sensory neuron projections from ipsilateral DRGs to dorsal column nuclei (Nucleus gracilis/Nucleus cuneatus) and one of the most investigated systems in spinal cord regeneration. It has long been appreciated that the intrinsic regenerative capacity of these neurons can be enhanced by lesions of their peripheral axons [123,124]. The ability to selectively label dorsal column sensory axons in the cervical spinal cord by peripheral injections of the transganglionic tracer cholera toxin ß into the sciatic nerve has facilitated detailed analyses of regenerative responses. This sensory system was also the focus of one of the first detailed studies examining the ability of NSC grafts to generate sensory neuronal relays across a lesion site. When a mix of rat glial-restricted and neuron-restricted precursors were grafted to rat dorsal column lesions at level C1, very close to the target nucleus (N. gracilis), strong evidence including electron microscopy, electrophysiology and stimulation-induced upregulation of c-fos expression indicated that grafted spinal progenitors can innervate target neurons and receive input from dorsal column sensory axons and transmit signals across a lesion site [67].

Evidence for synaptic connections between sensory axons and different types of NSC graft-derived neurons also comes from several more recent studies. In mice with dissociated E13 fetal NPC grafts in lesions at level C4, retrograde transganglionic labeling of DRG neurons was observed as low at the upper lumbar spinal cord [70]. As the tracing was initiated specifically from grafted cells expressing the receptor (TVA) for the pseudotyped rabies virus and the G helper protein for virus replication, these data strongly support the presence of monosynaptic sensory neuron to graft neuron connections. In addition, immunohistochemistry indicated the extension of nociceptive (CGRP+ and IB4+), proprioceptive and fine touch (NF200+ and calretinin+) projections into mouse NPC grafts [70]. Similar results were observed in rats grafted with NSCs generated from human ESCs (H9) that express the TVA receptor and G protein [110]. After helper dependent rabies virus injection into grafts in a dorsal column lesion, reporter gene expression was detected in DRG neurons as well Lamina III/IV-labeled neurons in the lumbar spinal cord. Although not further investigated, the latter might represent spinothalamic or other sensory projection neurons. Further support for functional connectivity between sensory projections and NPC grafts comes from studies examining in vivo calcium responses in grafted neurons. Following lower thoracic grafts of E12 NPCs from transgenic mice expressing the calcium reporter GCaMP6f, neuronal activity in response to light touch of the back, passive hindlimb movement or hind-paw pinch were clearly evident [112].

These data raise the question whether sensory innervation of graft-derived neurons is a random process or whether formation of synapses between sensory axon and graft-derived neurons is more selective and specific. In studies examining sensory axon regeneration into syngeneic E14 rat NPC suspension grafts in a dorsal column lesion, neurons expressing dorsal horn markers (Txl3, Lbx1, calretinin, calbindin) were not only clustered but also aligned in laminae-like patterns similar to the mature spinal cord [113]. This unexpected self-organization of grafted NPCs was also reflected in the density of sensory axon innervation. CGRP-labeled primary sensory projections were enriched in these dorsal clusters and capsacin injection into the skin resulted in higher c-fos labeling of neurons within calretinin-positive dorsal clusters. In addition, grafts from dorsal E14 spinal cord were more densely innervated by nociceptive peptidergic (CGRP-positive) axons than ventral spinal cord-derived NPCs [113]. The difference in neuronal composition between ventral and dorsal E14 spinal grafts was reflected in the depletion of TLX3-labeled sensory projection neurons in ventral grafts [93]. Similarly, in human fetal spinal cord NSC grafts to the injured non-human primate cord, CGRP penetration was also highly enriched in graft domains expressing TLX3 [93].

Taken together, the studies described above strongly suggest that mouse, rat and human NSC grafts are innervated by host sensory projections and therefore fulfill the first major prerequisite for functional sensory relays. There is also ample evidence for the second prerequisite, namely that primary sensory axons extending into NSC grafts form functional synapses, and that there is at least a preference if not some specificity in the graft-derived neuron population that receives sensory input. Regarding the third requirement for a sensory relay, namely innervation of relevant host sensory target nuclei by graft-derived axons, information is sparser, although NSC graft-derived axons extend into rostral direction in virtually every study. In immunodeficient rats with hiPSC-derived NSC grafts to C5 lateral hemisections, GFP-labeled graft-derived axons were found in dorsal column nuclei, medulla and midbrain and cerebellum, all sensory target nuclei [71]. Whether these axons made functional connections and whether sensory signals from areas caudal to the lesion are relayed across the lesion has only been shown in one rat study to date [67]. Whether these relays are mono- or polysynaptic, and whether there is any specificity of relay neurons relative to sensory modality, has not been analyzed and recovery of sensory function has only been observed electrophysiologically without behavioral correlates. Thus, the ability of NSC relays to influence sensory functional outcomes has not been explored in sufficient detail and merits further investigations.

## 8. Stem-Cell-Mediated Modulation of Pain after SCI

At level and below level pain after spinal cord injury is highly prevalent, in particular in SCI patients with incomplete injuries. An area of potential concern is the possibility that NSC transplantation worsens or induces pain symptoms either by inappropriate new connections or by influencing the pain circuitry in the surrounding host spinal cord. Most studies have not reported any potential sensory adverse effects of NSC grafts but specific functional tests that can detect nociceptive sensitization were not always conducted. On the contrary, using stem cells differentiated towards inhibitory lineages, amelioration of pain-like behaviors has been reported. While SCI-related neuropathic pain has many underlying mechanisms, spinal disinhibition (loss of inhibitory activity) resulting in hyperactivity of the pain circuitry plays an important role in the pathophysiology of pain. Thus, a localized increase in inhibitory neuronal activity or innervation by transplants of inhibitory interneurons could be an effective means of modulating pain.

Inhibitory neuronal progenitors obtained from the medial ganglionic eminence have been the focus of one set of studies. In animal models of peripheral neuropathies, grafting embryonic MGE-derived precursors into the lumbar spinal cord profoundly reduced pain-like behavior [125]. Transsynaptic tracing with wheat germ agglutinin showed that primary sensory neurons make synaptic connections to grafted neurons, and grafted neurons form synapses with host neurons in the spinal cord. Sensory stimulation also indicated increased c-fos expression in grafted neurons, further suggesting integration of grafted neurons in sensory circuits [125]. More recently, hESC-derived inhibitory telencephalic interneuron grafts into the mouse lumbar spinal cord after a T10 weight drop injury also showed a remarkable reduction of tactile allodynia and thermal hyperalgesia to levels observed in uninjured animals [126]. Functional benefits were also observed in studies using mouse ESCs differentiated towards inhibitory forebrain interneurons after a mouse contusion injury [127] and in studies with E14 rat cortical interneurons enriched for a GABAergic phenotype that were injected into the lumbar spinal after midthoracic clip compression injury [128]. Of note, all of these studies used cells without spinal identity, suggesting that a rostrocaudal identity does not impede integration into the spinal circuitry. Given the limited efficacy of pharmacologic pain relief, the localized enhancement of spinal inhibition by GABAergic stem cell-derived neurons might therefore be a promising strategy for clinical development.

## 9. Conclusions and Future Perspective

Despite the detrimental outcomes after SCI, there is no effective treatment to restore functional connectivity of the injured spinal cord. One promising strategy is to reconstruct the lesion site with neural stem/progenitor cell grafts (NSC/NPC) as transplanted cells can differentiate into neurons that may re-connect disrupted spinal neural circuitry. Recent studies from our and other groups support the concept of neuronal relay formation by transplant-derived neurons.

First, embryonic and pluripotent stem cell-derived NPCs can be transplanted into a lesion cavity and differentiate into neurons and glia. These developmentally early stage grafts integrate with the host tissue without generating a glial “scar” between host and graft [69], where grafted neurons extend their axons into the host spinal cord [72,73]. In turn, host spinal circuitry regenerates into the graft for graft–host connectivity. This reciprocal connection between transplanted neurons and host neural circuitry has the potential to constitute a new neuronal relay for descending motor and ascending sensory projections.

Second, recent work not only demonstrates successful regeneration of both descending supraspinal motor axons and ascending sensory axons into NSC/NPC transplants, but also the formation of functional synaptic connections. Corticospinal optogenetic stimulation in the motor cortex showed a clear calcium response in graft-derived neurons in the spinal cord, indicative of action potentials initiated by host motor circuitry [112]. Transsynaptic rabies virus tools further identified connections between NPC grafts and host circuitry (Figure 5A,B). Finally, stimulation of the hindpaw by pinch or touch of the lower back evoked fluorescent calcium responses in NPC grafts placed into a lesion cavity [112].

Third, host axons growing into these NSC/NPC grafts within the lesion cavity appear to have at least some specificity. Regenerating motor circuits extended their axons into the graft and formed synapses with premotor interneurons and motor neurons, avoiding sensory interneuron clusters within the graft [111]. In turn, ascending sensory circuits extended their axons towards sensory interneurons within the graft [93,113]. This indicates that host circuitry naturally finds appropriate synaptic targets within the stem cell graft, a critical component for the formation of a new functional circuit.

Fourth, NSC/NPC grafts support functional improvements after injury. NSC/NPCs with spinal identity placed into clinically relevant models of SCI resulted in functional improvement in hindlimb [72,110] and forelimb function [69,129] after injury and this improvement was sustained over several weeks.

Although the above studies support the role of NSC/NPC grafts as a potential therapeutic intervention for SCI, meaningful functional improvements in the clinic will likely require additional advances. While a large proportion of corticospinal axons penetrate into NSC/NPC grafts in the lesion site, most of these axons are located within the first 1 mm of the rostral host/graft interface. Intensive rehabilitation can strengthen and even reshape newly formed circuitry to promote functional outcomes after injury as rehabilitation can strengthen synapses [130], lead to the formation of new dendritic spines [131], and stabilize new networks [132]. Previous work has demonstrated that rehabilitation training and particularly task-specific rehabilitation treatment alone [133,134] or in combination with other therapeutic interventions [135,136] can improve skilled forelimb functional recovery. Thus, rehabilitation is likely to shape novel circuits formed by NSC/NPCs to result in more pronounced functional improvements.

Finally, potential adverse effects associated with NSC/NPC transplantation need to be addressed before clinical translation, including possible over-growth or tumor formation and transplant-induced pain. Consistent and reproducible protocols for the production of a sufficiently large number of cells under defined conditions have to be generated. Potency assays and cellular characterization with minimum acceptance criteria need to be established, and in vivo dose–response curves for efficacy and toxicity have to be completed. Parallel progress in the generation and transplantation of neuronal subpopulations most relevant for the formation of neuronal relays may further enhance the formation and efficacy of novel neuronal circuits. Thus, additional preclinical studies are warranted to support and improve the safety and efficacy of NSC/NPC therapy. Progress over the next years is likely to advance neural stem cells for the generation of neuronal relays in SCI towards clinical trials.
